# Comparative oral health status of two pre-Hispanic archaeological sites in Costa Rica

**DOI:** 10.3389/froh.2026.1753557

**Published:** 2026-03-05

**Authors:** Norberto F. Baldi, José Manuel Fernández-Chaves, Ana Cecilia Ruiz-Imbert, Daniela Infante-Herrera, Kevin Ibarra-Valverde, Karol Ramírez

**Affiliations:** 1 Biological Anthropology Laboratory, University of Costa Rica, San José, Costa Rica; 2 Faculty of Dentistry, University of Costa Rica, San José, Costa Rica; 3 School of Statistics, University of Costa Rica, San José, Costa Rica

**Keywords:** biocultural adaptation, Central America archaeology, dental pathology, oral-health indicators, pre-Hispanic Costa Rica

## Abstract

**Background/purpose:**

Dental and periodontal indicators provide key biocultural information for reconstructing diet, health, and cultural practices in ancient populations. This study compares oral health profiles from two pre-Hispanic Costa Rican sites located in distinct ecological settings: Palo Blanco (600–1,200 AD, Tempisque lowlands) and Rodríguez (800–1,550 AD, Central Highlands).

**Methods:**

Macroscopic dental data were recorded for 11 individuals (seven from Palo Blanco site and four from Rodríguez site). Variables assessed included dental caries, calculus, antemortem tooth loss (AMTL), periodontal state (CEJ–AC), dental wear, periapical lesions, malocclusions, and intentional dental modifications. Statistical analyses included Fisher's exact tests for categorical comparisons, Wilcoxon rank-sum tests for periodontal measurements, Wilson 95% confidence intervals, and Bonferroni corrections for multiple testing. To evaluate whether AMTL followed a directional pattern across ecological settings, we applied a Cochran–Armitage Trend Test for ordered proportions, using highland, intermontane, and lowland contexts as ordinal categories. We also analyzed 136 isolated teeth from the studied populations.

**Results:**

Although overall caries prevalence did not differ between Palo Blanco and Rodríguez, the anatomical patterning of lesions revealed clear contrasts: buccolingual and root caries were concentrated in the lowland Palo Blanco population, whereas occlusal caries was more frequent in the highland Rodríguez sample. Heavier supragingival and subgingival calculus deposits were detected in Palo Blanco across all scoring systems. AMTL displayed the most pronounced disparity, occurring in 85.7% of Palo Blanco adults but in none from Rodríguez (*p* = 0.015). Periodontal disease followed the same trend, with significantly greater CEJ–AC distances at Palo Blanco. When viewed alongside the intermediate frequencies reported, the two populations form a coherent ecological gradient, from the comparatively healthy dentitions of the cool highlands to the more inflamed oral environments of the tropical dry lowlands.

**Conclusions:**

Variation in periodontal disease, AMTL, and caries across the two sites reflect differing ecological conditions, food-processing practices, and dietary regimes. These results underscore the interpretive value of dental indicators for understanding biocultural adaptation in pre-Hispanic Costa Rica and provide a comparative baseline for future interdisciplinary studies incorporating isotopic, microwear, and biomolecular evidence.

## 1 Introduction

Human skeletal and dental tissues constitute an essential repository of information for reconstructing the biocultural dynamics of past populations. Teeth offer exceptional analytical value due to their resistance to taphonomic alteration and their capacity to preserve microscopic and macroscopic signals of diet, pathology, and behavior throughout the life course. Their structural durability makes them indispensable for evaluating caries, calculus, periodontal disease, non-carious lesions, and tooth wear, conditions shaped by both physiological stress and culturally mediated practices (1–4). Across the Americas, and especially in regions where faunal and botanical remains are fragmentary or poorly preserved, dental tissues often provide the most direct evidence of dietary change, food-processing technologies, and broader biocultural adaptations (5, 6).

In Costa Rica, the potential of dental anthropology remains underutilized. Bioarcheological research is uneven, with many osteological analyses confined to unpublished theses, isolated case studies, or technical reports lacking comparative depth. As Rojas and Murillo (7) observe, ongoing methodological limitations, particularly limited statistical rigor, weak alignment between research questions and collected data, and the lack of long-term archaeological projects, continue to impede the development of robust archaeological and bioarcheological frameworks in Costa Rica. Furthermore, preservation varies markedly between regions: the acidic soils and high rainfall of the Central Highlands and Caribbean watershed often limit skeletal survivorship, while the drier, more stable depositional conditions of the Pacific lowlands, particularly the Gran Nicoya cultural region, have yielded more substantial archaeological collections (8–10). These taphonomic and methodological disparities have constrained the development of regional models of oral health, diet, and disease.

Against this backdrop, several Costa Rican archaeological sites offer valuable opportunities for reconstructing ancient health and subsistence. In this study, we compare two archaeological populations from the Palo Blanco and Rodríguez sites. In the discussion section, we further contextualize our results by comparing them with data from a third archaeological site in Costa Rica, Agua Caliente. Although each site is located in a distinct ecological setting, they are broadly contemporaneous, dating to approximately 800–1,200 CE. Collectively, these sites represent tropical dry forest lowlands (Palo Blanco), montane highlands (Rodríguez), and an intermontane valley system (Agua Caliente).

Agua Caliente, located in the Valle del Guarco, is a large and archaeologically complex site with a long occupation sequence extending from the Pavas period into the Cartago period (ca. 300 BCE–1,550 CE). The site includes causeways, stone-faced platforms, and constructed terraces that articulate public spaces, residential areas, and ceremonial architecture, reflecting a high degree of spatial planning (11). Excavations have documented stratified domestic deposits, workshop areas, and funerary contexts exhibiting variability in mortuary treatment and access to prestige goods, patterns consistent with a hierarchically organized sociopolitical system (11, 12).

Although dental analyses from Agua Caliente remain limited to a relatively small number of individuals (13), the site's scale, architectural complexity, and long occupational history make it an important comparative reference for assessing regional variation in oral health and subsistence practices. Importantly, the individuals included in the present comparative sample were recovered from standardized stone cist burials with few associated offerings, a funerary pattern commonly interpreted as corresponding to non-elite social segments in Costa Rican archaeology (13). This relative social homogeneity reduces the likelihood that internal status differences account for the intermediate oral health profile observed at Agua Caliente and supports its interpretation as a biocultural outcome shaped primarily by its intermontane ecological context.

The Rodríguez archaeological site, located in Llano Grande de Cartago at approximately 1,400–1,600 m a.s.l., represents a highland settlement situated in a cooler, wetter montane environment characterized by volcanic soils and high annual precipitation. Excavations conducted by the Museo Nacional de Costa Rica indicate that Rodríguez was occupied during the Cartago period (ca. 800–1,200 CE), a time associated with diversified horticulture and the development of complex chiefdom systems in the Central Highlands (14).

In contrast, the Pacific lowland archaeological site of Palo Blanco (ca. 600-1,200 CE); (15), located in the lower Tempisque Basin, occupies an ecologically distinct tropical dry forest setting marked by pronounced seasonality, mosaic wetlands, and long-standing human use of lacustrine and riverine resources. These environmental conditions, documented since early naturalist observations (16) and later synthesized in regional ecological and biogeographical studies (17, 18), shaped pre-Columbian subsistence strategies centered on floodplain productivity, aquatic fauna, and the opportunistic exploitation of seasonally available resources (19). Despite its archaeological relevance of its funerary contexts, Rodríguez and Palo Blanco remains largely underrepresented in published bioarcheological research, making its inclusion essential for understanding regional variability in pre-Hispanic oral health.

The present study addresses this gap by generating new macroscopic dental data from Palo Blanco and Rodríguez and comparing them with published frequencies from Agua Caliente (13). These three populations occupy markedly different ecological zones, from highly productive tropical dry forest–wetland environments in the northwest to intensively managed highland agricultural landscapes in the central region, providing a valuable framework for evaluating how environmental parameters and biocultural processes intersected to shape oral pathology in pre-Hispanic Costa Rica during the late period and in different ecological settings. Based on these contrasts, we hypothesize that the prevalence and severity of dental caries, calculus, periodontal disease (CEJ–AC), and antemortem tooth loss (AMTL) will differ across populations, and that indicators associated with chronic oral inflammation will show inter-site variation.

By integrating newly collected data with existing archaeological evidence, this study offers the first systematic and statistically grounded comparison of oral health across three Costa Rican populations. Although the sample size is necessarily small, reflecting the limited preservation typical of Costa Rican archaeological contexts, the use of appropriate non-parametric statistical tests (e.g., Fisher's Exact Test, Wilcoxon Rank-Sum Test, and the Cochran–Armitage trend test) ensures analytical reliability and minimizes biases associated with small datasets. These findings contribute to broader discussions of biocultural diversity and adaptation in Lower Central America and establish a foundation for future interdisciplinary research combining dental anthropology, paleoenvironmental reconstruction, and biomolecular approaches.

## 2 Materials and methods

### 2.1 Study design and ethical compliance

This study followed a comparative cross-sectional design and was reported in accordance with the Strengthening the Reporting of Observational Studies in Epidemiology (STROBE) guidelines (20), adapted here for bioarcheological applications. All analyses relied exclusively on macroscopic examination of existing osteological collections. No destructive sampling or chemical preparation was performed, and therefore no special regulatory approval was required under the policies of the National Museum of Costa Rica (OFI-MNCR-DG-400-2024). Access to collections was authorized by the Biological Anthropology Laboratory of the University of Costa Rica. All procedures adhered to conservation protocols and ethical standards for working with pre-Columbian human remains, emphasizing respect, non-invasiveness, and curation integrity.

### 2.2 Data extraction

A forensic dentist and oral pathologist (JMFC) examined four maxillae and seven mandibles from Palo Blanco and Rodríguez archaeological sites. The analysis included assessment of caries, periapical lesions, antemortem tooth loss (AMTL), dental calculus, attrition, malocclusions, and dental modifications. Periodontal disease was evaluated by measuring the distance from the cementoenamel junction to the alveolar crest (CEJ–AC) for each preserved tooth, with mean values calculated per maxilla and mandible.

A bioarcheological analysis by NFB established associations between maxillae and mandibles for two individuals from the Palo Blanco site. Due to the inability to pair the remaining maxillae with their corresponding mandibles, a comprehensive analysis of oral health status was conducted using the total tooth assemblage from each site to facilitate inter-site comparisons.

The study also examined 136 individual teeth: 70 from Palo Blanco (two excluded due to significant deterioration) and 66 from Rodríguez. Assessment included evaluation of caries, non-carious dental lesions (abrasion, erosion, and abfraction), dental attrition, and calculus deposits.

The state of preservation of dental and osseous materials were better at the Rodríguez archaeological site than at Palo Blanco, likely due to differences in environmental conditions that differentially affect skeletal remains.

### 2.3 Assessment of dental pathologies and other conditions in maxillae and mandibles

Caries assessment followed the criteria of Hillson (21). A lesion was recorded only when cavitation was macroscopically visible; enamel discoloration alone was not considered diagnostic unless cavitation was present beneath it. All teeth were examined under bright lighting using a dental probe. For each tooth, the presence, number, anatomical location (enamel, dentin, pulp), and affected surfaces (occlusal, buccal/lingual, interproximal, or root) were documented.

Periapical bone lesions were defined as osteolytic cavities caused by infection spreading through the root canal into surrounding alveolar bone. For each tooth position, the presence or absence of a perforating fistula or sinus tract at the apex was recorded. Antemortem tooth loss (AMTL) was diagnosed when the alveolar socket showed remodeling or resorption indicative of healing; sockets with no evidence of remodeling were classified as postmortem losses.

Dental attrition was documented using the Smith and Knight (22) tooth-wear index, which evaluates the loss of enamel and exposure of dentine across four surfaces (buccal, cervical, lingual, and occlusal–incisal). Scores ranged from 0 (no contour loss) to 4 (complete enamel loss, pulp or secondary dentine exposure, and defects exceeding 2 mm in depth). Attrition was also scored using Molnar's (23) eight-grade system, which categorizes wear from Grade I (no wear) to Grade VIII (roots only), allowing comparative evaluation across all tooth types.

Dental calculus was identified by its location, color, and surface morphology. Severity was classified using Brothwell's (24) criteria (none; slight; moderate; severe) and quantified following Knussman's scoring system as applied by Vodanović et al. (25, 26). Subgingival calculus, an indicator relevant to periodontal disease and tooth loss, was categorized as slight, moderate, or severe.

Periodontal disease was assessed through measurements of the cemento-enamel junction to alveolar crest (CEJ–AC) distance, following Gonçalves et al. (27). Measurements were taken at six standardized sites around each tooth (mesiobuccal, mid-buccal, distobuccal, mesiolingual/palatal, mid-lingual/palatal, distolingual/palatal) using a UNC-15 probe positioned parallel to the long axis of the tooth. A mean CEJ–AC value was calculated per tooth and then averaged by maxilla and mandible.

A ZEISS Stemi 305 stereo microscope with 5:1 zoom, was used to classify dental modifications according to the categories and styles established by Romero in 1958 (28). The system distinguishes three major groups—alterations to crown shape, modifications of the labial surface, and combined alterations—subdivided into seven styles (A–G), which include occlusal-edge filing, union bipartite angular reshaping, labial incisions, mineral inlays (e.g., jade, pyrite, turquoise, gold), asymmetrical patterns, and inlay–contour combinations. Additionally, the UNC-15 periodontal probe with millimeter markings, was used to determine the depth and width of the central notches.

For regional comparison, individual-level data from 24 individuals from the Agua Caliente cemetery were incorporated (13). Because the Agua Caliente dataset does not include tooth-level observations, inter-site comparisons with this assemblage were restricted to individual-level variables using the AMTL data.

### 2.4 Assessment of dental pathologies in individual teeth

Caries, non-carious lesions, dental attrition, and calculus assessment for individual teeth from both sites were recorded as described above (21–28).

### 2.5 Analytical framework and comparative design

A total of 70 individual teeth from Palo Blanco and 66 from Rodríguez were examined. All teeth were recovered as loose finds during excavation (i.e., not in anatomical position). Consequently, it is not possible to determine the minimum number of individuals represented by the 70 teeth from Palo Blanco or the 66 teeth from the Rodríguez site. Comparative analyses focused on variables consistently available across all sites: caries, calculus, AMTL, periapical lesions, and periodontal disease. Agua Caliente data was incorporated for individual-level comparisons of AMTL.

### 2.6 Statistical analysis

**Figure 1:**
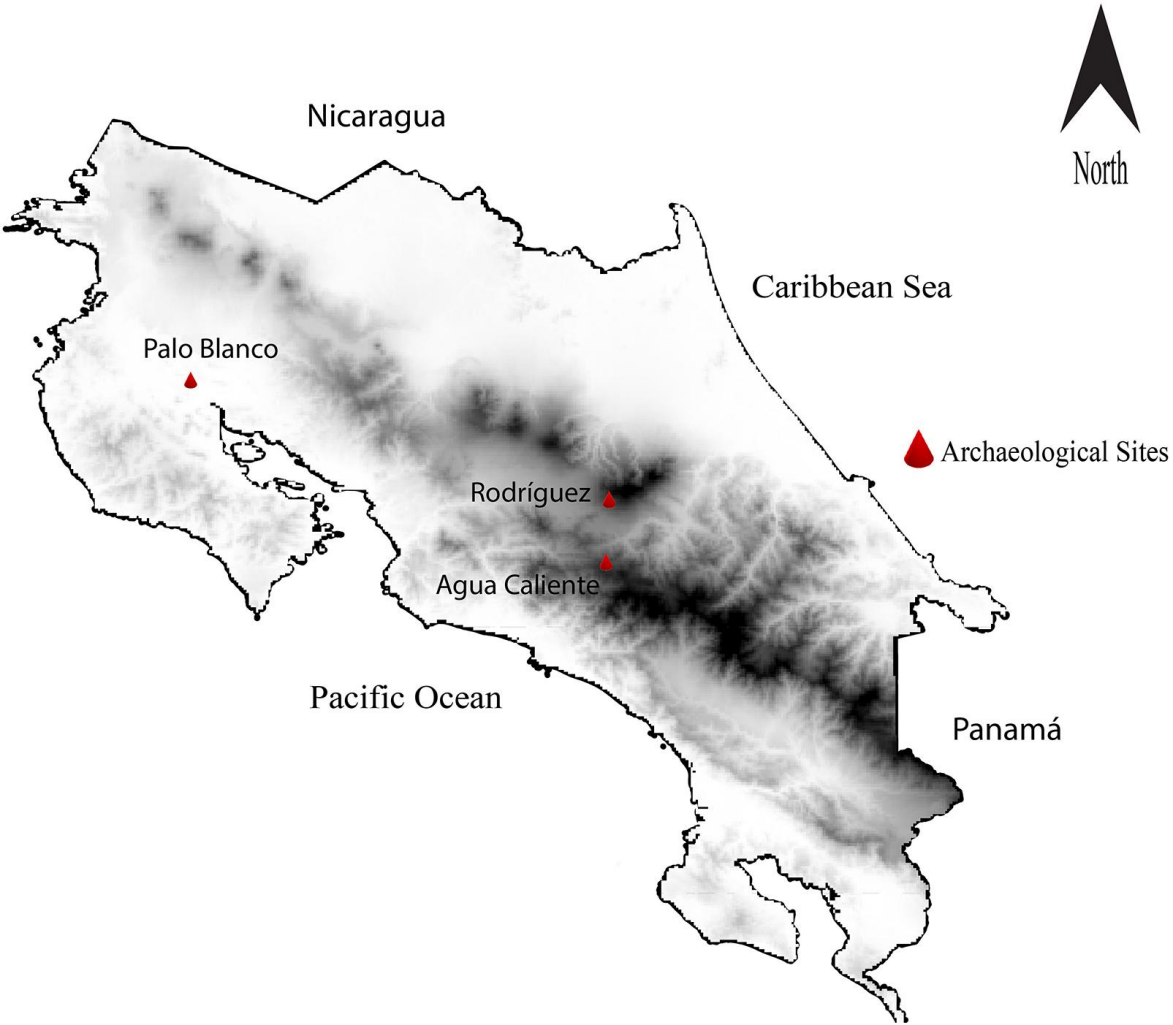
Geographic map of Costa Rica illustrating spatial relationships among the archaeological sites.

All statistical analyses were conducted in R 4.4.0 (R Core Team, 2024). Given the small and uneven nature of archaeological dental samples, all procedures emphasized non-parametric and exact approaches to ensure robust inference. Categorical variables, including the presence or absence of caries, calculus, and AMTL, were evaluated using Fisher's exact test for pairwise inter-site comparisons, complemented by Wilson 95% confidence intervals to quantify uncertainty and assess overlap between populations. To mitigate inflated Type I error rates arising from multiple pairwise tests, Bonferroni-adjusted significance thresholds were applied following recommended protocols for small-n bioarcheological datasets. Periodontal differences between Palo Blanco and Rodríguez were assessed using the Wilcoxon rank-sum test because CEJ–AC measurements failed to meet assumptions of normality and variance homogeneity. To test broader biocultural hypotheses regarding directional variation across ecological zones, we incorporated a Cochran–Armitage trend test to evaluate whether AMTL frequencies followed a statistically significant ordinal gradient across the highland (Rodríguez), intermediate (Agua Caliente), and lowland (Palo Blanco) sites defined in the Introduction. Because these sites form an *a priori* ordered ecological sequence, we assigned ordinal scores of 0 (Rodríguez), 1 (Agua Caliente), and 2 (Palo Blanco), following recommended procedures for trend testing in categorical data. The analysis was performed in R 4.4.0 using the function Desc Tools: Cochran Armitage Test. This test assessed whether AMTL prevalence increased monotonically along the environmental gradient, providing a formal statistical evaluation of the proposed biocultural model. This trend analysis explicitly assessed whether oral pathology increased or decreased along an environmentally structured axis, providing a formal complement to pairwise comparisons. The geographic map (Figure 1) illustrating spatial relationships among the archaeological sites was produced with QGIS 3.34.

## 3 Results

### 3.1 Remaining dentition of maxillae and mandibles

Table 1 summarizes the distribution of caries of AMTL, periapical lesions, malocclusions, and non-carious lesions in the preserved dentition of maxillae and mandibles from Palo Blanco and Rodriguez archaeological sites. Fisher's exact tests were used to evaluate inter-site differences. For caries, *p*-values were contrasted against an *α* = 0.007 following Bonferroni adjustment. AMTL and periapical lesions were tested at *α* = 0.05, and malocclusions at *α* = 0.0125. No statistical tests were performed for dental gyroversion or non-carious lesions due to the absence of variability.

**Table 1:** Distribution of dentoalveolar pathologies: absolute and relative frequencies by archaeological site.

Dentoalveolar pathologies	Archaeological Site				*p*-value^a^
	Palo Blanco		Rodríguez		
Caries					
Enamel	5	(71.4%)	2	(50.0%)	0.576
Dentin	4	(57.1%)	2	(50.0%)	1.000
Pulp	3	(42.9%)	2	(50.0%)	1.000
Surface Occlusal	3	(42.9%)	2	(50.0%)	1.000
Surface Buccal-lingual	2	(28.6%)	1	(25.0%)	1.000
Surface Interproximal	2	(28.6%)	2	(50.0%)	0.576
Surface Root	3	(42.9%)	1	(25.0%)	1.000
AMTL	6	(85.7%)	0	(0.0%)	**0** **.** **015**
Periapical bone lesions	6	(85.7%)	3	(75.0%)	1.000
Malocclusions					
Overcrowding	2	(28.6%)	0	(0.0%)	1.000
Gyro version	0	(0.0%)	0	(0.0%)	-
Labioversion	2	(28.6%)	0	(0.0%)	1.000
Linguoversion	2	(28.6%)	0	(0.0%)	1.000
Eruption abnormalities	0	(0.0%)	2	(50.0%)	0.364

ANTM, antemortem tooth loss.

Bold *p* values denote statistical significance.

^a^ Fisher's exact test used for statistical analysis.

Occlusal wear was broadly similar between sites. Table 2 summarizes dental attrition based on the Smith and Knight index. Statistical significance was evaluated using Bonferroni-adjusted alphas: 0.0167 for the Smith and Knight scores (0.05/3), 0.0063 for Molnar's ranks (0.05/8. Variables marked “–” showed no variation and were not tested. Overall, both populations exhibited similarly advanced occlusal wear, especially on posterior teeth, and no significant inter-site differences were detected, indicating comparable masticatory stress and dietary abrasiveness between the two communities.

**Table 2:** Distribution of dental attrition: absolute and relative frequencies by archaeological site.

Dental attrition	Archaeological Site				*p*-value^a^
	Palo Blanco		Rodríguez		
Smith & Knight tooth					
Wear index scoring					
Score 1	2	(28.6%)	3	(75.0%)	0.242
Score 2	7	(100.0%)	3	(75.0%)	0.364
Score 3	4	(57.1%)	1	(25.0%)	0.546
Score 4	0	(0.0%)	0	(0.0%)	-
Molnar					
Grade I	0	(0.0%)	2	(50.0%)	0.109
Grade II	2	(28.6%)	3	(75.0%)	0.242
Grade III	5	(71.4%)	3	(75.0%)	1.000
Grade IV	4	(57.1%)	0	(0.0%)	0.194
Grade V	3	(42.9%)	1	(25.0%)	1.000
Grade VI	2	(28.6%)	1	(25.0%)	1.000
Grade VII	2	(28.6%)	0	(0.0%)	0.491
Grade VIII	0	(0.0%)	1	(25.0%)	0.364

^a^ Fisher's exact test used for statistical analysis.

At the individual level (Table 3), both populations exhibited high frequencies of calculus, and no significant differences were detected after multiple-testing corrections. This contrast with the tooth-level findings likely reflects the limited number of adult individuals per sample.

**Table 3:** Distribution of dental calculus: absolute and relative frequencies by archaeological site.

Dental Calculus	Archaeological Site				*p*-value^a^
	Palo Blanco		Rodríguez		
Brothwell (24)					
No calculus	5	(71.4%)	2	(50.0%)	0.576
Slight	6	(85.7%)	4	(100.0%)	1.000
Moderate	5	(71.4%)	2	(50.0%)	0.576
Severe	4	(57.1%)	1	(25.0%)	0.546
Quantity of Dental Calculus					
(Knussman)					
1	3	(42.9%)	3	(75.0%)	0.546
2	6	(85.7%)	4	(100.0%)	1.000
3	4	(57.1%)	2	(50.0%)	1.000
4	3	(42.9%)	1	(25.0%)	1.000
5	0	(0.0%)	1	(25.0%)	0.364
6	4	(57.1%)	0	(0.0%)	0.194
Subgingival Calculus					
Slight	6	(85.7%)	2	(50.0%)	0.491
Moderate	6	(85.7%)	2	(50.0%)	0.491
Severe	5	(71.4%)	2	(50.0%)	0.576

^a^ Fisher's exact test used for statistical analysis.

Periodontal disease was assessed using CEJ–AC distances, differed significantly between sites. As shown in Table 4, Palo Blanco individuals exhibited greater recession (mean = 5.02 mm) than those from Rodríguez (mean = 3.06 mm), a difference confirmed by a Wilcoxon rank-sum test (*p* < 0.05). This pattern aligns with the heavier calculus loads observed at Palo Blanco.

**Table 4:** Average CEJ-AC by archaeological site.

Dental parameter	Archaeological Site		*p*-value^a^
	Palo Blanco	Rodríguez	
CEJ-AC	5.023	3.056	**0** **.** **024**

*p*-value was contrasted against an alpha of 0.05 for significance.

CEJ-AC, distance from the cementoenamel junction to the alveolar crest.

Bold *p* values denote statistical significance.

^a^ Wilcoxon rank sum exact test used for statistical analysis.

### 3.2 Results of individual teeth

All dental specimens were recovered *ex situ*, lacking anatomical association. As a result, the minimum number of individuals represented by the assemblages from Palo Blanco (*n* = 70) and Rodríguez (*n* = 66) cannot be determined. Overall caries prevalence was moderately higher at Palo Blanco (34.3%) than at Rodríguez (25.8%). When lesions were evaluated by surface category (Table 5), significant inter-site differences emerged. Buccolingual enamel/dentin caries were more common at Palo Blanco (17.1% vs. 4.5%; *p* = 0.002), and root caries occurred exclusively at this site (12.8% vs. 0%; *p* = 0.003). Other lesion types showed no significant contrasts after Bonferroni correction.

**Table 5:** Prevalence of dental caries and non-carious lesions by archaeological site.

Dento-alveolar pathologies	Archaeological Site				*p*-value^a^
	Palo Blanco		Rodríguez		
Caries					
Enamel	26	(37.1%)	33	(50.0%)	0.166
Dentin	24	(34.3%)	17	(25.8%)	0.350
Pulp	7	(10.0%)	3	(4.5%)	0.327
Surface Occlusal	9	(12.9%)	21	(31.8%)	0.012
Surface Buccal-lingual	12	(17.1%)	1	(1.5%)	**0** **.** **002**
Surface Interproximal	12	(17.1%)	8	(12.1%)	0.473
Surface Root	9	(12.9%)	0	(0.0%)	**0** **.** **003**
Non carious dental lesions					
Abrasion	3	(4.3%)	0	(0.0%)	-
Erosion	0	(0.0%)	0	(0.0%)	-
Abfraction	1	(1.4%)	0	(0.0%)	-

Statistical significance was assessed using an alpha of 0.05. Bonferroni adjustment was applied to control for multiple comparisons, resulting in an adjusted alpha level of 0.007.

Bold *p* values denote statistical significance.

^a^ Fisher's exact test used for statistical analysis.

Patterns of dental wear are presented in Table 6 using the Smith and Knight and Molnar wear indices. Under the Smith and Knight system, both populations exhibited substantial posterior occlusal wear, with no significant inter-site differences (*p* = 0.488). Molnar's ordinal grading similarly revealed comparable distributions of wear severity across sites (*p* = 0.371). Together, these results indicate broadly similar masticatory loads and abrasive dietary conditions among individuals from Palo Blanco and Rodríguez.

**Table 6:** Distribution of Smith & Knight and Molnar Wear index scores by archaeological site.

Dental attrition	Archaeological Site				*p*-value^a^
	Palo Blanco		Rodríguez		
Smith & Knight tooth					
Wear index scoring					0.488
Score 1	12	(20.7%)	18	(29.5%)	
Score 2	32	(55.2%)	32	(52.5%)	
Score 3	14	(24.1%)	11	(18.0%)	
Score 4	0	(0.0%)	0	(0.0%)	
Molnar					0.371
Grade I	4	(6.9%)	10	(15.4%)	
Grade II	10	(17.2%)	10	(15.4%)	
Grade III	19	(32.8%)	17	(26.2%)	
Grade IV	13	(22.4%)	16	(24.6%)	
Grade V	11	(19.0%)	9	(13.8%)	
Grade VI	1	(1.7%)	0	(0.0%)	
Grade VII	0	(0.0%)	3	(4.6%)	
Grade VIII	0	(0.0%)	0	(0.0%)	

All *p*-values were contrasted against an alpha of 0.05 for significance.

^a^ Fisher's exact test used for statistical analysis.

Dental calculus accumulation varied substantially between populations. As shown in Table 7, Brothwell's classification revealed significantly heavier calculus at Palo Blanco (*p* = 0.0008). Knussmann's scoring showed an even more pronounced difference (*p* = 0.0001), and subgingival calculus also differed significantly between sites (*p* = 0.0031). These results indicate consistently heavier calculus deposition in the Palo Blanco population.

**Table 7:** Distribution of dental Calculus: absolute and relative frequencies by archaeological site.

Dental Calculus	Arqueological Site				*p*-value^a^
	Palo Blanco		Rodríguez		
Brothwell (24)					**0** **.** **0008**
No calculus	19	(27.1%)	11	(16.7%)	
Slight	34	(48.6%)	17	(25.8%)	
Moderate	13	(18.6%)	24	(36.4%)	
Severe	4	(5.7%)	14	(21.2%)	
Quantity of Dental Calculus					
(Knussman)					**0** **.** **0001**
1	20	(28.6%)	10	(15.2%)	
2	31	(44.3%)	14	(21.2%)	
3	5	(7.1%)	3	(4.5%)	
4	7	(10.0%)	10	(15.2%)	
5	4	(5.7%)	18	(27.3%)	
6	3	(4.3%)	11	(16.7%)	
Subgingival Calculus					**0** **.** **0031**
Slight	27	(55.1%)	27	(46.6%)	
Moderate	7	(14.3%)	21	(36.2%)	
Severe	15	(30.6%)	10	(17.2%)	

All *p*-values were contrasted against an alpha of 0.05 for significance.

Bold *p* values denote statistical significance.

^a^ Fisher's exact test used for statistical analysis.

**Figure 2:**
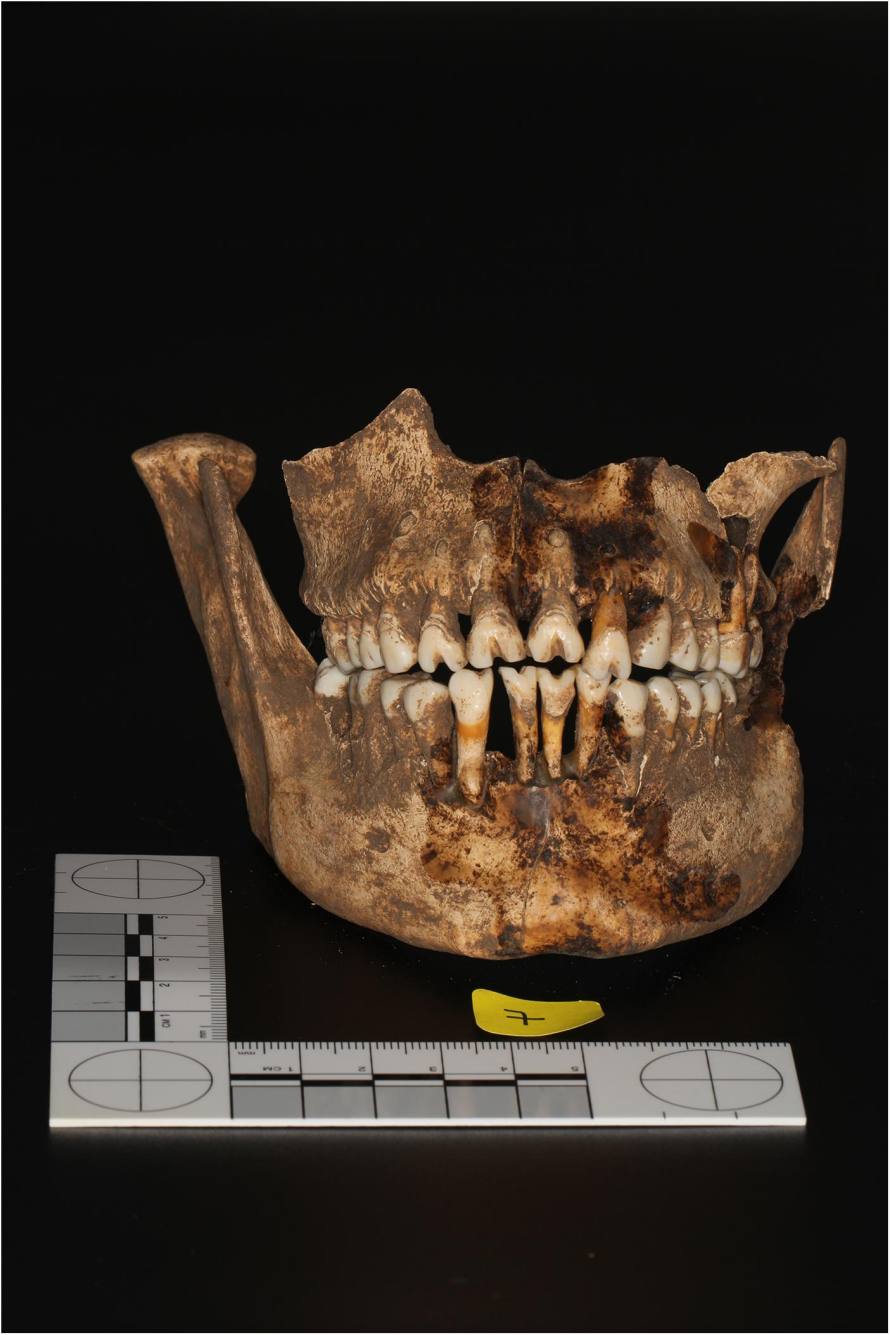
An adult individual from Palo Blanco exhibited intentional dental modifications affecting both the maxillary and mandibular incisors.

**Figure 3:**
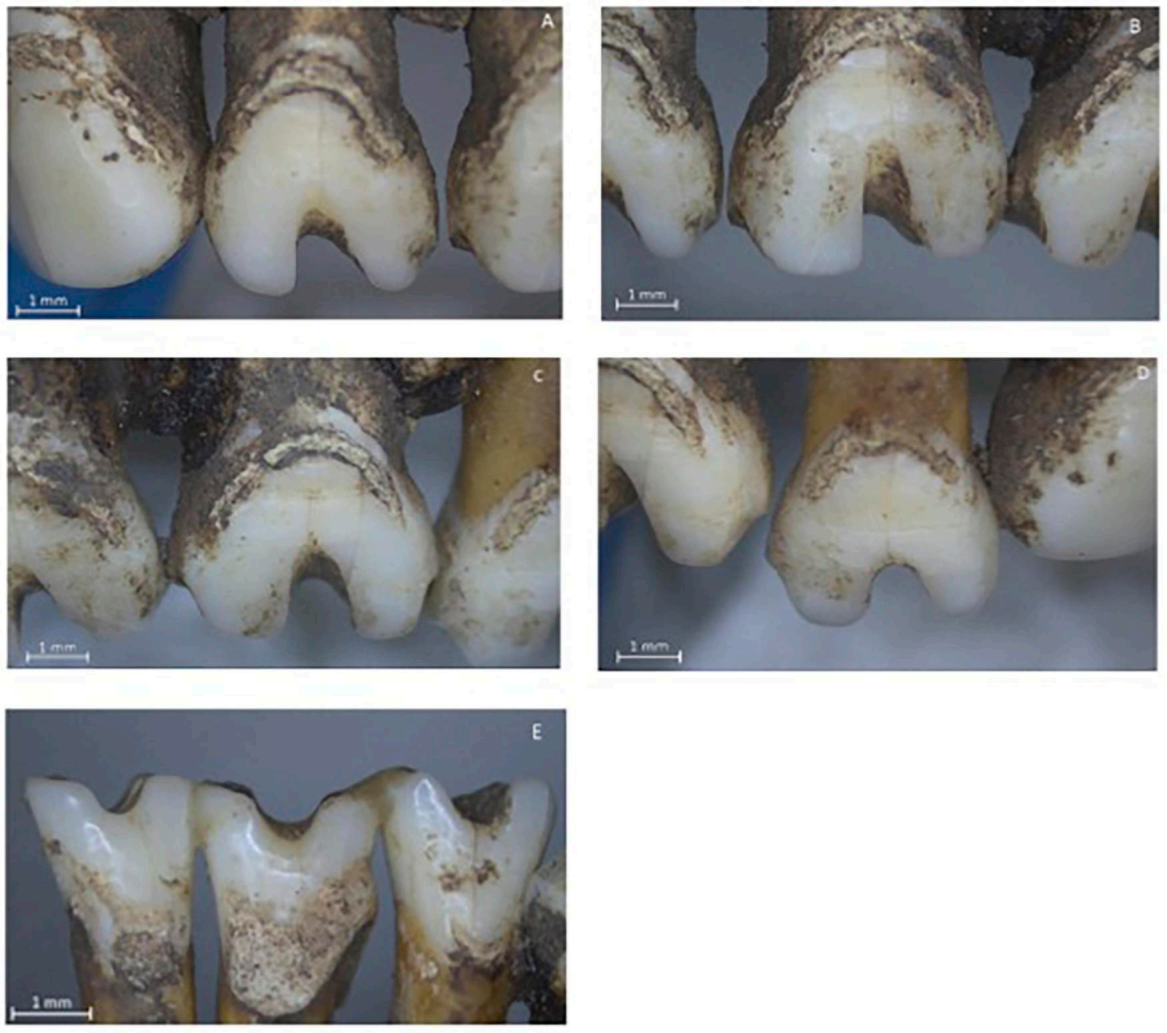
Dental modifications observed in one individual from the Palo Blanco archaeological site. Panels **(A,B,D)** show Type F1 dental modifications in teeth 12, 11, and 22. Panel **(C)** illustrates a Type F10 dental modification in tooth 21. Panel **(E)** shows remnant lower incisors exhibiting Type A1 dental modifications. Scale bars on panels **(A–E)**: 1 mm.

One adult individual from Palo Blanco exhibited intentional dental modifications affecting both the maxillary and mandibular incisors (Figure 2). The sex of the individual could not be determined. Examination under a stereomicroscope revealed a Type A1 modification on the mandibular incisors, according to the classification proposed by Romero (28). Type A1 refers to a horizontal cut across the incisal edge of the anterior teeth. The same individual also exhibited Type F1 and Type F10 dental modifications. Figure 3 shows the dental modifications. Panels (A), (B), and (D) show Type F1 dental modifications in teeth 12, 11, and 22. Panel (C) illustrates a Type F10 dental modification in tooth 21. Panel (E) shows remnant lower incisors exhibiting Type A1 dental modifications.Type F1 refers to a groove or filing on the labial (vestibular) surface of the tooth. Type F10 modification is characterized by a more complex pattern on the labial surface. The notches ranged in depth from 1.5 to 3 mm and in width from 2 to 3 mm. No other individuals in the sample showed evidence of cultural dental modification. The occurrence of A1, F1, and F10 modifications in this individual is consistent with previously documented practices in pre-Columbian Costa Rican populations, where these three types are reported as the most common forms of incisor modification (29, 30).

### 3.3 Cochran–armitage trend test for proportional differences

Antemortem tooth loss varied sharply across the three populations. As presented in Table 8, AMTL was highest at Palo Blanco (85.7%; 6/7 adults), intermediate at Agua Caliente (30%; 3/10), and lowest at Rodríguez (0%; 0/4). Fisher's exact tests indicated significant contrasts between Palo Blanco and Rodríguez (*p* = 0.015) and between Palo Blanco and Agua Caliente (*p* = 0.0498). A Cochran–Armitage trend test using ordered ecological scores detected a significant positive linear trend (Z = 2.93; *p* = 0.0034), indicating that AMTL increases systematically along the highland-to-lowland gradient.

**Table 8:** Antemortem tooth loss (AMTL) by individual across the three archaeological sites.

Site	Individuals with AMTL	Total, Adults (≥20 yrs)	% AMTL
Palo Blanco	6	7	85.7%
Agua Caliente	3	10	30.0%
Rodríguez	0	4	0.0%

Values correspond to adult individuals presenting clear evidence of remodeled alveoli indicating antemortem tooth loss. Agua Caliente values derive from Salazar-Camacho (13), (Table 4, p. 13). Pairwise Fisher tests: Palo Blanco vs. Rodríguez: ***p*** **=** **0.015,** Palo Blanco vs. Agua Caliente: ***p*** **=** **0.0498,** Rodríguez vs. Agua Caliente: ***p*** **=** **0.5055** (ns). among adults across three pre-Hispanic Costa Rican sites arranged along an ecological gradient (highland Rodríguez → intermediate Agua Caliente → lowland Palo Blanco). A Cochran–Armitage trend test detected a significant positive linear trend (Z = 2.93, *p* = 0.0034).

## 4 Discussion

The comparative analysis of dentoalveolar pathologies across Palo Blanco, Rodríguez, and Agua Caliente reveals a patterned and ecologically structured distribution of oral health indicators in pre-Hispanic Costa Rica. Although individuals from Palo Blanco and Rodríguez exhibited the substantial occlusal wear characteristic of pre-industrial populations, the severity and anatomical distribution of pathological conditions varied systematically along the environmental gradient represented by these communities. When Agua Caliente is included in the comparison, interpretation is restricted to antemortem tooth loss (AMTL), the only oral health indicator directly comparable across all three sites. In this framework, AMTL follows a clear gradient, with the highest frequencies in the lowland Palo Blanco population, intermediate values at Agua Caliente, and the lowest frequencies in the highland Rodríguez sample. This structured pattern aligns with long-standing anthropological observations that oral health reflects the interplay of ecology, subsistence practices, and inflammatory processes (2, 21, 31, 32). Similar regionally patterned variability has been documented worldwide, where differences in food-processing technologies, diet composition, and local environmental conditions shape the prevalence of caries, periodontal disease, and AMTL (33–36). Together, these comparative frameworks support the interpretation that the Costa Rican populations examined here were embedded in distinct ecological niches that produced divergent oral microbiomes and biocultural trajectories of dental health.

AMTL exhibited the clearest inter-site divergence, with the highest frequencies in Palo Blanco (Pacific lowlands), lowest in Rodríguez (Central Highlands), and intermediate values in Agua Caliente (Guarco Basin). The Cochran–Armitage trend test confirmed this as a significant directional pattern, demonstrating that chronic oral deterioration increased from highland to lowland settings. AMTL is a cumulative indicator of lifelong exposure to caries, periodontal inflammation, and mechanical and infectious stresses (32, 37). Its distribution therefore reflects persistent differences in oral environments rather than random variation.

The significantly greater CEJ–AC distances observed in Palo Blanco reinforce this interpretation, as larger CEJ–AC values are widely recognized as markers of periodontal disease and alveolar bone loss in archaeological populations (38). The combined pattern of elevated AMTL, greater CEJ–AC, and higher frequencies of root caries at Palo Blanco suggests longstanding inflammatory conditions affecting the supporting tissues of the dentition.

Although overall caries prevalence did not differ significantly between Palo Blanco and Rodríguez when categories were aggregated, the anatomical distribution of lesions revealed meaningful contrasts. Buccolingual enamel/dentin caries and root caries were significantly more common in Palo Blanco, a pattern closely associated with gingival recession and sustained plaque accumulation at the cervical margin (3, 21, 33, 39). These findings are consistent with the heavier periodontal burden documented in this lowland population, where chronic periodontal inflammation likely exposed root surfaces and facilitated demineralization (38). In contrast, occlusal caries was significantly more common in Rodríguez. This pattern is consistent with differences in occlusal surface exposure, wear dynamics, and microenvironmental conditions on the crown surface rather than disparities in periodontal inflammation (34, 35). Together, these patterns underscore that caries distributions reflect multiple interacting processes, including diet, wear, morphology, and soft-tissue inflammation, and therefore cannot be attributed to a single causal factor (2, 31).

The presence of intentional dental modifications on a single individual at the Palo Blanco archaeological site includes Type F1 and F10 alterations on the upper incisors, along with a Type A1 modification on the lower incisors, based on the classification established by Romero (28). These types are well-documented and widely distributed across Mesoamerica and Lower Central America, and their occurrence at Palo Blanco aligns with this broader cultural tradition. In northwestern Costa Rica, particularly within the Gran Nicoya region, Type A1 modifications—horizontal cuts along the incisal edge of anterior teeth, have been recorded at multiple sites. A study by Valerio and Chavarría (29) found A1 to be the most prevalent type among individuals excavated at Jícaro, Nacascolo, and La Cascabel, comprising over 43% of all documented cases. While F1 and F10 were not identified in that particular study, subsequent research by Barzuna and Núñez (30) reported the presence of all three types (A1, F1, and F10) at La Cascabel, El Silo, and Jícaro, pointing to a more diverse pattern of dental modification in the region. The appearance of these styles at Palo Blanco reinforces the idea that dental modification served not only as a widespread tradition, but also as a meaningful expression of identity, social status, or cultural affiliation throughout Mesoamerica (40, 41).

In broader Mesoamerican and Isthmo-Colombian contexts, differences in caries patterns have been linked to variability in carbohydrate processing, food texture, and the mechanical properties of subsistence staples (6, 42). While this study does not employ isotopic or micro botanical evidence to infer specific diets, the anatomical patterning of lesions observed at Palo Blanco is compatible with long-term inflammatory processes rather than any single dietary factor.

Dental calculus formation showed the most pronounced inter-site differences aside from AMTL. Individuals from Palo Blanco exhibited substantially higher supragingival and subgingival calculus accumulation, as well as higher Knussmann index scores, than those from Rodríguez. These findings suggest meaningful differences in mineralization tendencies, salivary chemistry, or chronic inflammatory load—factors known to vary with environmental and biocultural parameters, including water mineral content, airborne particulates, and dietary abrasiveness (43–45). Additional support comes from clinical and bioarcheological studies indicating that subgingival calculus formation and cervical plaque retention are intensified in contexts with sustained periodontal disease (31, 38). While these ecological influences are plausible, the present dataset cannot directly identify the underlying mechanisms, and interpretations remain inferential.

By comparison, Rodríguez individuals exhibited higher proportions of calculus categories associated with moderate mineralization, consistent with cooler and wetter highland environments that may reduce supragingival mineral deposition relative to drier lowland contexts (43). That calculus and CEJ–AC exhibit parallel cross-site rankings strengthen the interpretation that periodontal disease and mineralization processes, rather than masticatory stress, underlie much of the observed inter-population variation.

Despite strong differences in periodontal and carious indicators, occlusal wear patterns did not differ significantly between Palo Blanco and Rodríguez. Both populations exhibited heavy, multi-plane wear consistent with abrasive diets, the widespread use of stone grinding technology, and a reliance on coarse or fibrous food resources documented throughout pre-Hispanic Costa Rica (8, 46). The absence of significant differences in occlusal wear, even as periodontal and AMTL patterns vary markedly, illustrates the multifactorial nature of oral health: masticatory stress does not necessarily covary with inflammatory or infectious processes (2).

Although fewer variables were available for Agua Caliente, the intermediate values documented for AMTL and caries are consistent with the broader ecological trend observed in this study. Archaeologically, Agua Caliente occupies an intermontane valley context characterized by diversified horticulture, sociopolitical complexity during the Cartago period, and access to a broader spectrum of subsistence resources than either high-elevation or lowland dry forest contexts (11, 12).

Taken together, the results show that oral pathology in pre-Hispanic Costa Rica followed predictable and ecologically structured patterns. Lowland tropical dry forest populations such as Palo Blanco experienced the greatest burden of periodontal inflammation, calculus formation, and antemortem tooth loss (AMTL), while highland populations such as Rodríguez exhibited comparatively good periodontal health despite similar degrees of mechanical wear. Intermontane valley populations, represented by Agua Caliente, displayed intermediate pathological profiles. These differences are unlikely to reflect fundamentally distinct cultural traditions, as the three populations are broadly contemporaneous and embedded within the same late pre-Hispanic cultural horizon of Costa Rica, sharing core technological and subsistence practices characteristic of the Isthmo-Colombian area (9, 47). Instead, archaeological evidence indicates that these communities occupied contrasting ecological and settlement contexts—Pacific lowlands, Central Highlands, and intermontane valleys—which structured how shared cultural behaviors were expressed in daily life (11, 14, 15).

These findings highlight the interpretive value of integrating multiple dental indicators: caries, calculus, periodontal disease, occlusal wear, and AMTL, to reconstruct biocultural processes shaping oral health. They also reinforce broader Central American patterns in which oral pathology varies with ecological setting, subsistence strategies, and long-term oral microbiome dynamics (2, 48). Future research integrating stable isotope analysis, dental microwear texture analysis, sediment chemistry, and biomolecular evidence from calculus will further refine interpretations of subsistence, mobility, and oral microbiomes in ancient Costa Rican populations and deepen our understanding of biocultural diversity in the Isthmo-Colombian area.

## 5 Conclusions

This study provides the most detailed comparative assessment to date of dentoalveolar health across three pre-Hispanic Costa Rican populations situated in distinct ecological settings (ca. 800–1,200 AD). The results reveal that oral pathology followed a consistent environmental gradient: individuals from the lowland tropical dry forest site of Palo Blanco exhibited markedly greater calculus accumulation, more advanced periodontal disease, and the highest frequencies of antemortem tooth loss, whereas the highland Rodríguez population showed comparatively healthy periodontal conditions despite similarly heavy occlusal wear. Agua Caliente, located in an intermontane valley, displayed intermediate pathological profiles that align with its environmental and cultural practices. These findings indicate that oral disease patterns were shaped by long-term interactions among ecological variables, subsistence behaviors, and chronic inflammatory processes rather than by mechanical loading alone.

By integrating multiple dental indicators, caries distribution, CEJ–AC distances, calculus severity, wear indices, and AMTL, this study demonstrates the value of multivariate approaches for reconstructing biocultural processes and clarifying the independent contributions of masticatory stress, mineralization patterns, and periodontal disease. Although the small sample sizes, particularly for Agua Caliente, limit fine-grained interpretations, the cross-site consistencies documented here establish an initial framework for understanding how local environmental conditions influenced oral health in ancient Costa Rican populations.

These results highlight the need for broader regional datasets, especially given the scarcity of comparable dental studies in the Isthmo-Colombian area. Future research integrating stable isotope analysis, dental microwear texture analysis, microbotanical residues, sediment chemistry, and biomolecular evidence from dental calculus will further refine reconstructions of diet, mobility, and oral microbiome dynamics, contributing to a deeper understanding of biocultural diversity and ecological adaptation in ancient Central America. Additionally, future studies should explore differences in oral health across social classes, gender groups, and differential access to resources, in order to test hypotheses regarding ecological and health-related inequalities, as proposed in this study. Such integrative approaches will be essential to understanding the interplay between identity, environment, and social structure in pre-Columbian populations.

## Data Availability

The original contributions presented in the study are included in the article/Supplementary Material, further inquiries can be directed to the corresponding author.
